# Stress State Classification Based on Deep Neural Network and Electrodermal Activity Modeling

**DOI:** 10.3390/s23052504

**Published:** 2023-02-23

**Authors:** Floriana Vasile, Anna Vizziello, Natascia Brondino, Pietro Savazzi

**Affiliations:** 1 Department of Electrical, Biomedical and Computer Engineering, University of Pavia, 27100 Pavia, Italy; 2 Brain and Behavioral Sciences Department, University of Pavia, 27100 Pavia, Italy

**Keywords:** wearable, EDA, emotion recognition, skin conductance, NN, deep learning

## Abstract

Electrodermal Activity (EDA) has become of great interest in the last several decades, due to the advent of new devices that allow for recording a lot of psychophysiological data for remotely monitoring patients’ health. In this work, a novel method of analyzing EDA signals is proposed with the ultimate goal of helping caregivers assess the emotional states of autistic people, such as stress and frustration, which could cause aggression onset. Since many autistic people are non-verbal or suffer from alexithymia, the development of a method able to detect and measure these arousal states could be useful to aid with predicting imminent aggression. Therefore, the main objective of this paper is to classify their emotional states to prevent these crises with proper actions. Several studies were conducted to classify EDA signals, usually employing learning methods, where data augmentation was often performed to countervail the lack of extensive datasets. Differently, in this work, we use a model to generate synthetic data that are employed to train a deep neural network for EDA signal classification. This method is automatic and does not require a separate step for features extraction, as in EDA classification solutions based on machine learning. The network is first trained with synthetic data and then tested on another set of synthetic data, as well as on experimental sequences. In the first case, an accuracy of 96% is reached, which becomes 84% in the second case, thus demonstrating the feasibility of the proposed approach and its high performance.

## 1. Introduction

Nowadays, wearable and implantable technologies in healthcare have become a reality with the progress in engineering technologies, and will promote next generation healthcare to enable personalized medicine through real-time physiological monitoring [1,2].

Wearable sensors are non-invasive and more comfortable, and have already been employed for stress detection. In [3], the authors introduce a new and unobtrusive wearable monitoring device based on electrodermal activity (EDA) to be used in health-related computing systems. The acquired EDA of a subject is used to detect his/her calm/distress condition, placing the wearable device on the wrist of the subject to allow continuous physiological measurements.

Since autistic people can face problems tolerating invasive electrodes [4], wearable sensors may be extremely useful for estimating emotional state changes in non-verbal people [5]. According to that, in [4], the authors assess tactile perception in early childhood autism by means of psychophysical approaches.

Some reliable and available technologies are magnetoencephalography (MEG), functional magnetic resonance imaging (fMRI), electroencephalography (EEG), and heart rate variability (HRV). The main drawbacks are cost and hindrance; thus, the need for a non-intrusive sensor arises.

Electrodermal Activity (EDA) is one of the promising and non-invasive technologies for detecting people emotional state variations. EDA was already observed from the late 1880s [6], but only in the last four/five decades has the research intensified, due mainly to the technology progress and miniaturization [7]. Despite that, only a few commercial wearable devices include this feature. The fact that only the autonomous nervous system is responsible for EDA has been studied [8], so that the measured signal is used mainly to assess distress, anxiety, and attention. Recently, EDA measurements were also exploited for other applications, from pain detection to dementia monitoring [7].

Following this reasoning, we aim to use the detected EDA signal to infer emotional state variations in autistic people. This could be useful since, in an overwhelming or overstimulating environment, autistic people may face meltdowns, which are a loss of behavioural control [9]. Knowing this information in advance could enable caregivers, or the autistic person himself, to take appropriate action to prevent such crises, thus enabling a higher quality of life.

Usually, the EDA signal is analyzed by extracting some features related to arousal and stress states such as the number of peaks per minute [6] or the skin conductive response (SCR) [10], considering both their time and frequency analysis. However, often these studies on EDA signal collection and analysis are difficult to replicate [10]. As better detailed in Section 2, existing works for EDA classification usually employ machine learning methods, such as [11], leveraging on features extraction and available experimental datasets.

In this work, a different approach is proposed: a deep neural network (DNN) is used with a synthetic data model to generate the sequences for training the network. The features are thus implicitly extracted and engineered by the network and the need for a high number of data points is fulfilled by the synthetic data without using the available datasets, which often show some criticalities such as the short length of the recorded sequences. This problem is well known, as seen in [12], and synthetic data represent one of the solutions proposed in the literature. In this way, the proposed approach ensures an inexhaustible source of data, thus overcoming the difficulty in finding available datasets, as well as the high number of samples required for artificial intelligence approaches. In addition, the obtained synthetic data can be considered well annotated, as stress details are set as parameters. Moreover, they are ground-truth error-free and annotated consistently, while it is still difficult to improve realism and close the gap between synthetic and experimental data.

Going into more detail, starting from [13,14], we developed a synthetic data model, based on the usual decomposition of the signal in a slow varying skin conductance level (SCL), called baseline, and the SCR, which contains more neuronal spikes due to the sympathetic activity related to the stressful condition. The parameters of this model were set by considering what is reported in literature [6,13,14] and our previous data exploration on other experimental sequences [15]. The obtained synthetic data are used to train a DNN, and experimentally recorded data are employed to test the network classifier. Very good performance is obtained with an accuracy of around 84%, which becomes 96% when testing the classifier with another set of synthetic data. Very good performance is obtained with an accuracy of 96% when testing the classifier with another set of synthetic data, while around 84% on experimental data. The synthetic test data were generated in the same way, i.e, through the same model, as the original training and validation data. In this way, the problem of lacking a large amount of training data can be overcome and overfitting effects can be avoided at the same time. In addition, the proposed algorithm could be easily implemented in a smart band device.

The rest of the paper is organized as follows: Section 2 presents previous works on EDA signal classification and the strategies to overcome the lack of experimental data. Section 3 details the data model to generate synthetic data, which are used in the training phase of the neural network described in Section 4. Experimental results are shown in Section 5 and Section 6 closes the paper with some summarizing conclusions.

## 2. Related Work

In the last several decades, EDA has been used to understand the nervous system activity. The sweat glands are innervated by the sympathetic nervous system, which is involved in emotions regulation. The activity of sweat glands is triggered by postganglionic sudomotor fibres that are also responsible for thermoregulation. For this reason, the EDA signal is often decomposed into two different overlying signals: one is the SCL and the other is the SCR. The former is due to the presence of sweat on the skin, mainly for thermoregulation purposes, while the latter is related to emotional arousal [6].

These two components, also named tonic and phasic, respectively, can be decomposed and analyzed using different techniques [13,14,16]. The phasic component is related to arousal and stress states, and is characterized by the presence of peaks corresponding to the onset of stimuli. After obtaining this component, usually, a peak extraction is performed [16] to understand the arousal level. In several works, the SCR activity level has been assessed by counting the number of peaks over time, such as [6,16].

Identification of emotional states can be viewed as a classification task [17], and it has been demonstrated that it is possible to infer human emotional activities from EDA measurements without the need for other physiological signals [17].

A common step in classifying emotional states is data annotation [18], which is usually performed manually or with a self-assessment manikins (SAM) questionnaire, as in [17]. The SAM proposes different intermediate levels of choices from ‘happy’ to ‘unhappy’ state, from ‘excited’ to ‘calm’ state, and from ‘controlled’ to ‘in control’ state.

For automatic classification, often deconvolution techniques are employed as a first step to analyze EDA sequences [15]. Then, after a feature extraction step, classification-based solutions are employed to classify the emotional state [3,19,20].

In [3], the skin conductivity response (SCR) is estimated by means of discrete deconvoluton and time-frequency extracted features. A statistical analysis of the features was performed by means of an analysis of variance (ANOVA) test and then an SVM was used for classification.

Since deconvolution techniques are usually based on parametric models, in [15], some of the authors of this work have investigated the possibility to improve the extraction of features related to arousal emotional states by designing an adaptive blind deconvolution filter. It is demonstrated that adaptive filtering can be used to deconvolve the measured EDA sequences by extracting the SCR peaks, which should carry the information about the subject’s activity level.

Learning methods are usually used for classification, such as a support vector machine with recursive feature elimination (SVM-RFE) [11], a convolutional neural network (CNN) [21], a principal component analysis (PCA) followed by SVM [22], and a radial basis function kernel (SVMR) with multilayer perceptron (MLP) and random forest (RF) [23].

Differently, some methods were developed that do not require a separate step for feature extraction since they leverage on neural network (NN), such as [24,25], and the proposed solution.

In Table 1, different works are reported, highlighting the achieved classification accuracy and the publishing year. It is clear that the comparison is made between different techniques that are used on EDA sequences, which are recorded in different ways. These methodologies were used, in fact, on actually different online available datasets. Every dataset represents a different experiment and a different way of labelling the data, even though they are still EDA signals.

**Figure 1:**
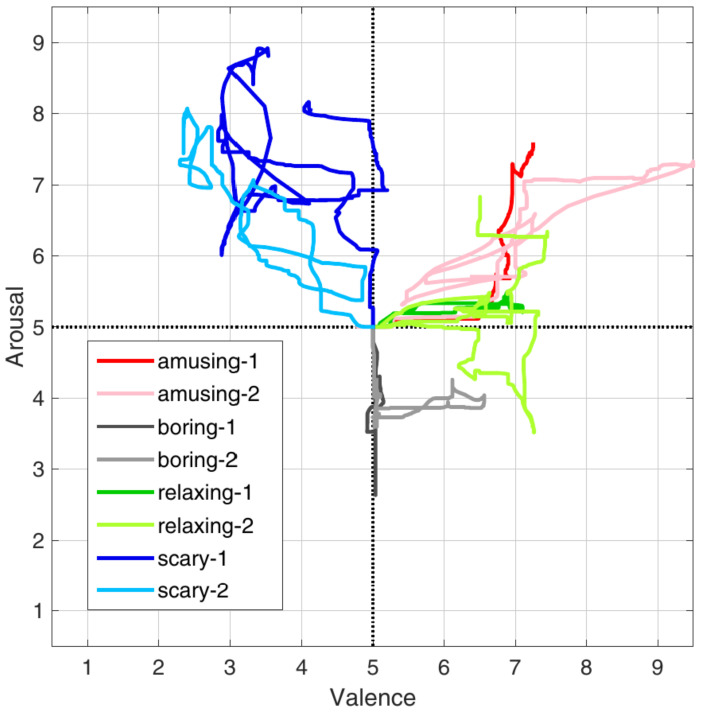
The way the data are annotated and the difference between arousal and valence in [26].

In the above-mentioned table, the accuracy reported is on the ones taken from the studies, which categorize the arousal and not the valence of the emotion (if they were both present in the paper, the former was chosen). The difference between valence and arousal is based on the fact that the arousal can be described as the intensity of the emotion, while the valence refers to the fact that it can be seen as positive or negative, like happiness vs. sadness. A better understanding can be achieved by looking at Figure 1, where it presents how they labelled the experimental data in [26].

In [24], a Long Short-Term Memory Neural Network (LSTM NN) was used to predict stress using EDA data and a regression was made to predict the forthcoming stress level. The results from [15] can be replicated using an NN with LSTM layers, using it for the deconvolution of the sequences obtaining the peaks. Indeed, the problem, related to how to use this information, still remains and a classificator should still be used.

LSTM are also used in Auto Encoders (AEs), which are employed to obviate the need of anomalous data, which are very often lacking. This is the case of the considered scenario, since it is not possible to trigger a meltdown to record the EDA signal, representing the abnormal signals. However, it may happen that an AE reconstructs not only signals similar to those used for training, but also abnormal signals that have never been seen before by the AE [25].

A training mechanism was proposed to avoid such issue [25]. Indeed, this AE behaviour may pose a challenge for emotional state classification from EDA signals. Specifically, assuming to use only neutral signals for training, if the AE can reconstruct both neutral and stress signals, it is not possible to select features to distinguish between them in order to classify the emotional state. However, this may be due to the nature of EDA signals since the two types of signals show the same shape and differ only in the number of peaks per minute.

**Figure 2:**
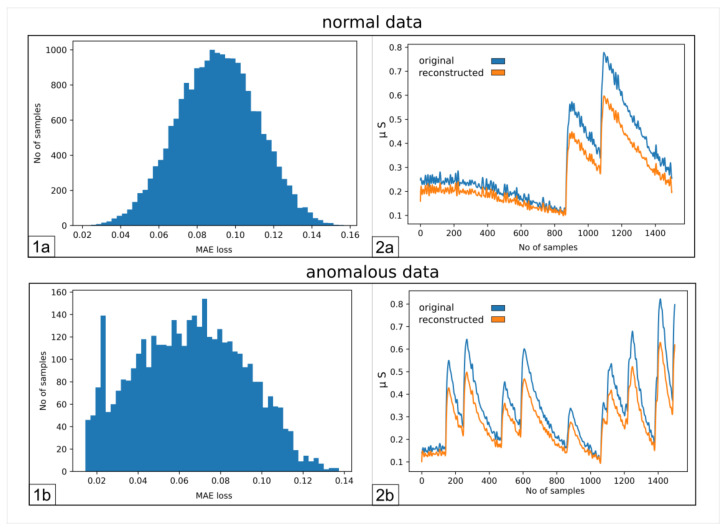
(**1a**) Number of samples vs. Mean Absolute Error (MAE) obtained when reconstructing the normal sequences; (**1b**) as 1a, but for anomalous data; (**2a**) example of an original signal (blue) and the corresponding reconstructed one (orange); (**2b**) same as (**2a**), but for anomalous data.

Figure 2 illustrates the results that we obtained employing an AE, trained only on neutral samples and then tested on both neutral and active signals. Figure 2(1a,1b) represent the distribution of the error over all the sequences during the reconstruction performed by the AE.

**Figure 3:**
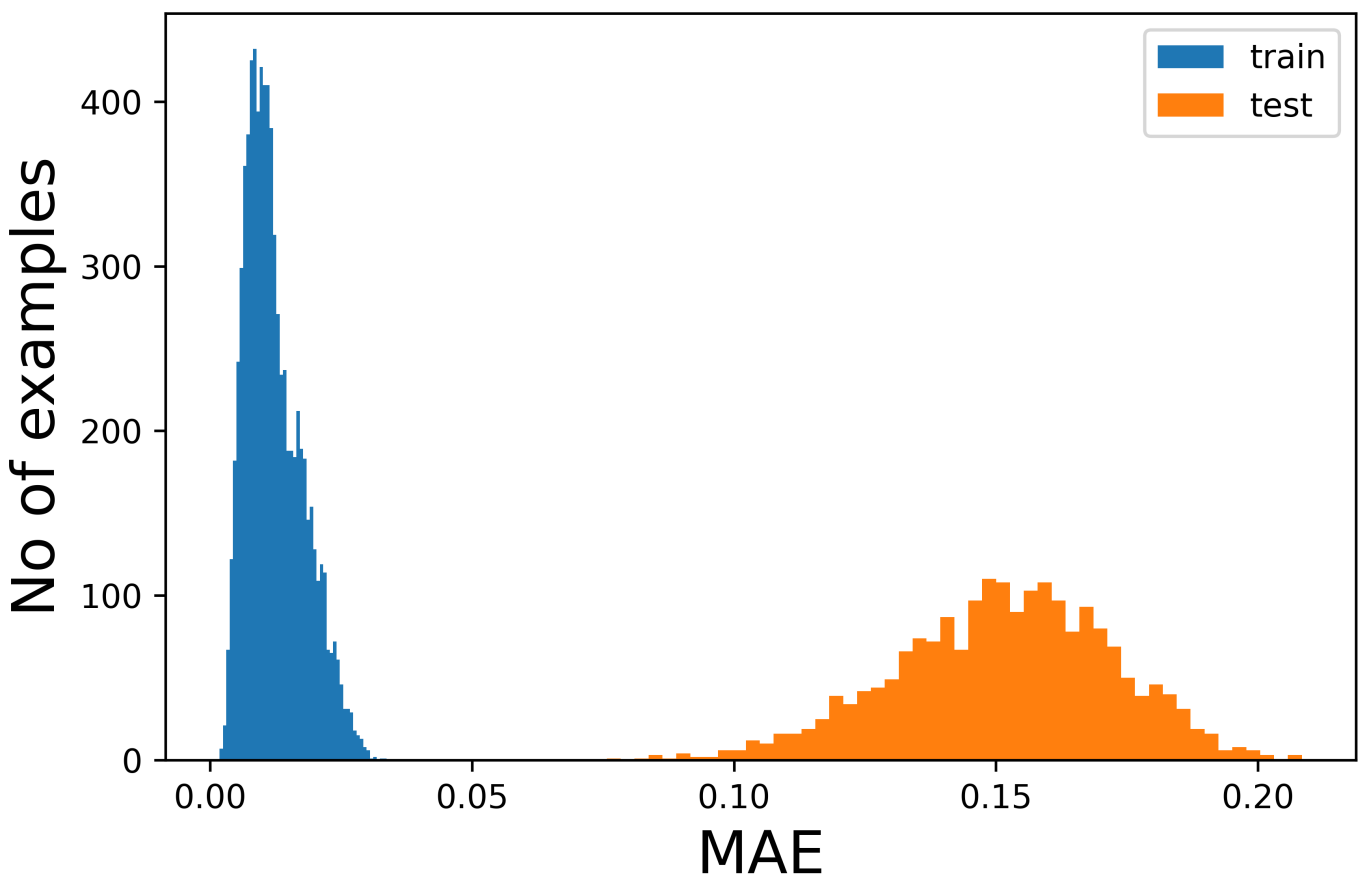
Number of samples vs. Mean Absolute Error (MAE) obtained when reconstructing the normal sequences and the anomalous data. In this case, which is ideal, the classification would be easy and the threshold for the MEA would be around 0.05.

**Figure 4:**
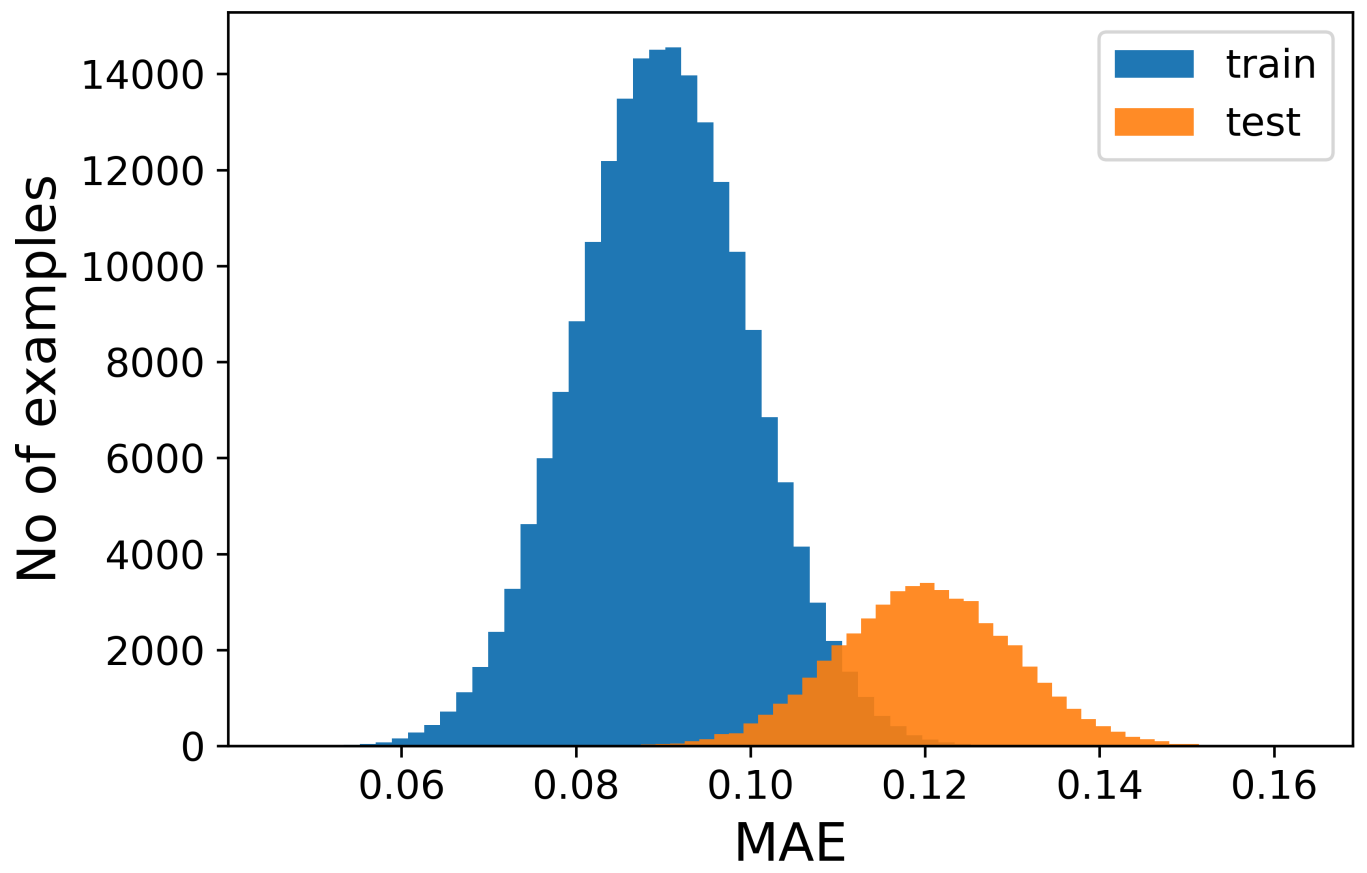
Number of samples vs. Mean Absolute Error (MAE) obtained when reconstructing the normal sequences and the anomalous data. This case represents the most common situation, in which there is an overlap between the two different error distributions. The classification is still possible, but not accurate, since the overlap does not allow for establishing a threshold to separate the two different kinds of sequences. It is still possible to choose a value for the error, which would implicate that some of the normal sequences would be seen as anomalous and vice versa.

**Figure 5:**
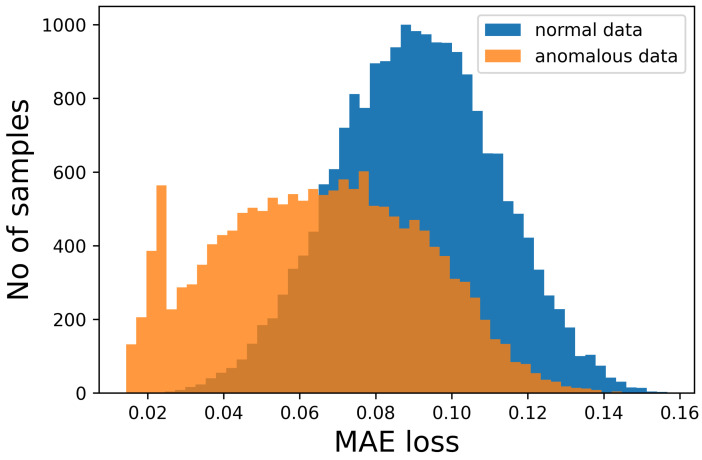
Number of samples vs. Mean Absolute Error (MAE) obtained when reconstructing the normal sequences and the anomalous data. This is what happens with our sequences, as seen also in Figure 2. In this figure, it is possible to see, in more detail, how the two distributions almost completely overlap, preventing the discrimination between the two.

The figures show that it is not possible to discriminate the anomalous data with respect to the normal ones because the bell-shaped curves of the error distribution overlap completely. What could be desirable is shown in Figure 3, where the two sets of data (train and anomalous) are easy to distinguish. In Figure 4, it is possible to observe what typically happens when using this technique. It is common, in fact, that the autoencoder makes some mistakes on normal data and/or reconstructs some of the anomalous ones well, leading to some superimposition of the calculated MEA. In this case, the threshold would be chosen to optimize the results, analyzing the problem and understanding if it is more acceptable to categorize anomalous data as normal or the contrary. In Figure 5, it is possible to see our case, in which the superposition does not allow for distinguishing the two cases at all.

Thus, it is not feasible to set a threshold for the Mean Absolute Error (MAE) to distinguish the two types of sequences. If the errors made on the anomalous sequences were higher than the other sequences, it would be possible to set an error threshold above which the signal would be categorized as anomalous. In this way, the sequences would be reconstructed, the error calculated and then compared to this threshold, allowing the discrimination.

For this reason, we propose to generate EDA synthetic data for both normal and anomaly conditions and use them for training a DNN.

## 3. Synthetic Data Model

In this section, we present the model used to generate training data for the NN devoted to stress state classification, which will be discussed in Section 4. This model was used also in [15], but, for this work, it was improved to take into account some variability in the data.

Electrodermal activity signal refers to the variation of the skin electrical conductance, and is made up of two components: a phasic one, which is event related and impulsive, and a tonic response that is slowly varying. Moreover, an additive, white noise component is added to take into account thermal noise effects.

The main goal of this work is to exploit an EDA model in order to train a classification algorithm able to recover the emotional state of a subject after collecting real-life experimental data. To this end, the pseudo-random variability of the EDA model is used to generate a sufficient number of training sequences for the NN subsequently used for classifying real data signals.

The usual way to classify the emotional arousal is to count the number of peaks [6], and this is made possible, as shown in many works such as [14], after a subtraction procedure, in which the slowly varying part is removed. In [15], we followed a similar method, using an adaptive deconvolution filter for estimating the spike-driven signal.

The model from [15] assumes to have a discrete-time EDA signal with a sampling frequency equal to $f_{s}$. At every time step, a peak can arise, following the human physiology variations, for which the pulse train is sparse. The main model is built considering the slowly varying signal, i.e., the baseline, $b\left(nT_{s}\right)\equiv b\left(n\right)$ with $T_{s}=1/f_{s}$, a Gaussian noise v(n), while the phasic component is modeled as a sparse impulse signal x(n) convolved with an impulse response h(n):

(1) y(n)=h(n)∗x(n)+b(n)+v(n)

where h(n) is usually defined according to the Bateman model [14]:

(2) $$h\left(n\right)=g\left(e^{\frac{-nT_{s}}{\tau_{1}}}-e^{\frac{-nT_{s}}{\tau_{2}}}\right),$$

in which the suitable time constants $\tau_{1}$ and $\tau_{2}$ are set as follows. The baseline is modeled as a slow varying signal added to the phasic one. In this way, the overall signal is the sum of the baseline, the Gaussian noise, and the convolution of *x* with the sweat response signal *h*, as in (1).

In this work, the model was further improved, with respect to [15], to take into consideration variability across different situations and individuals and improve generalization, which is very important to avoid overfitting. The resultant sequences were still given by the sum of the previous three components, but the convolution is obtained using different filter parameters for each peak. In more details, the number of peaks is randomly generated following a uniform distribution, and considering the interval (1,5) for the neutral state and (6,20) for the active one.

**Figure 8:**
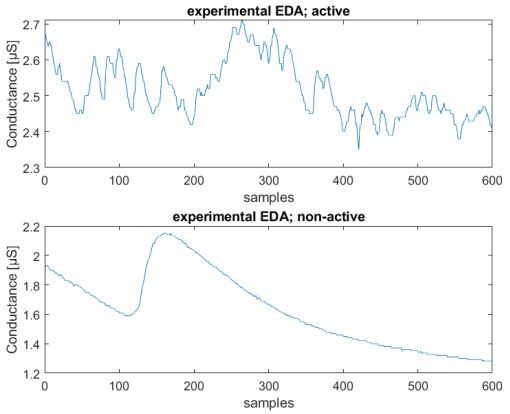
Example of experimental EDA signals: neutral sequence (**above**), active sequence (**below**); conductance [μS] vs. number of samples (total sequence length—2 min).

The constant time parameters $\tau_{1}$ and $\tau_{2}$ can vary respectively in the intervals (1,40) and (0.2,1) s, by randomly generating these filter parameters each time a peak occurs. In this way, we expect to obtain a higher agreement with experimental data and a better performance of the NN, avoiding at the same time overfitting phenomena. In Figure 8, a typical EDA signal is represented with a comparison between active and neutral sequences.

### 3.1. Data Preparation

The synthetic sequences were generated at a sampling frequency equal to 5 Hz, with a length of 600 samples that corresponds to a duration of 2 min, like the experimental data. The two model parameters $\tau_{1}$ and $\tau_{2}$, and the number of the peaks per minute were randomly generated as defined above.

**Figure 6:**
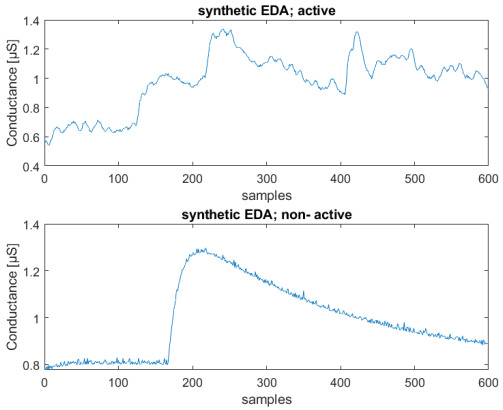
Example of active state (**above**) and neutral state (**below**) of synthetic signals; Conductance [μS] vs. number of samples (total sequence length—2 min).

In Figure 6, we can see an example of both neutral and active synthetic sequences that can be compared with the two experimental ones shown in Figure 8.

### 3.2. Experimental Data

**Figure 7:**
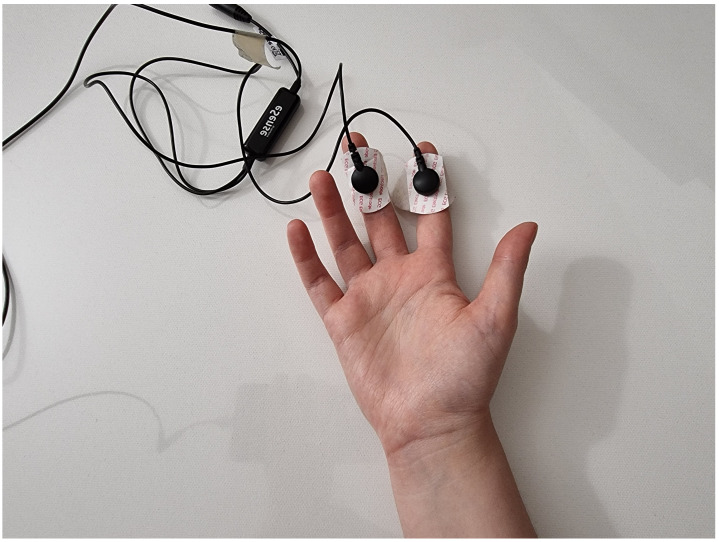
Electrodes placement.

The experimental data were recorded by means of a Mindfield eSense Skin Response [27], at a sampling frequency of 5 Hz. In Figure 7, an example of the placement of the electrodes is shown.

Two different tasks were performed during data recording: for the non-active sequences, the subject had to stay relaxed and avoid thinking about stressful situations or thoughts, while for the stressful one, the person had to stay on one leg or perform an isometric exercise, in order to physically emulate a very stressful situation, such as a meltdown, avoiding at the same time movement artefacts and preventing sweating due to thermoregulation.

The length of the sequences was set to a duration corresponding to 5 min, to avoid changes in emotional state if longer periods of time are considered. For instance, it is inherently difficult to stay in a relaxed state for a longer time, due to involuntary thoughts. The total number of recorded sequences is 80, and the obtained dataset is balanced, so that half of them are non-active, and half active. The final number of sequences, obtained using a window of 600 samples, is 320. These sequences were obtained with an overlap of 300 samples, as in [28]. Figure 8 shows an example of two active and non-active experimental recorded sequences.

## 4. Neural Network

In this work, a DNN is trained to classify EDA recorded signals. The main difference from previous works, such as [17], is that here we use a model for generating training data instead of employing experimental ones. This is actually due to the need of a huge number of sequential training sequences, useful for obtaining satisfactory results from DNN classification, while each of the datasets available in the literature contains only no more than 30 sequences [26,29]. The usage of synthetic data for training the NN makes this technique non-specific and not tuned only on a peculiar experimental case.

### Fully Convolutional Network

**Figure 9:**
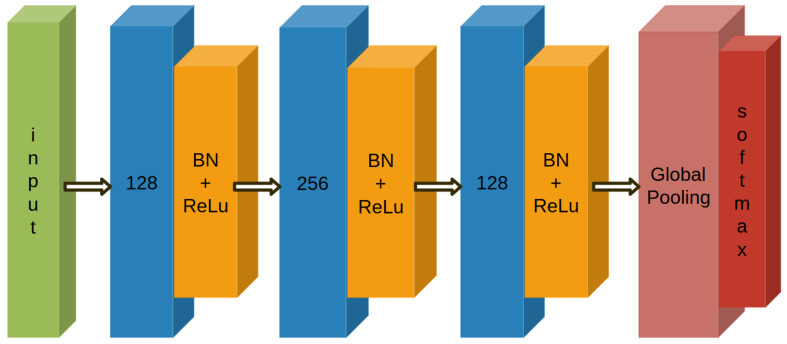
The net structure from [30].

The network used in this work is the one proposed in [30], where its performance is compared with the residual network (ResNet) and the multilayer perceptron (MLP) ones. The net structure can be seen in Figure 9. This network is able to implement features extraction without the heavy pre-processing usually required with other types of neural networks. This is very important, especially considering the everyday health monitoring by means of small and wearable devices, with limited computational resources. In this sense, the feature engineering is not performed, letting the algorithm find itself the contributing region of the data for each label.

In [30], it is possible to see how this net was tested on 44 UCR time series datasets [31]. There are four different metrics, three of which indicate that FCN reaches a higher performance than the other proposed nets.

#### Architecture and Training Options

The network is formed by a block of layers that are stacked onto each other. The basic layers that are used in each block are a convolutional, a batch normalization and rectified linear unit (ReLU) activation layers. As reported in [30], these blocks can be formalized as:

(3) $$\begin{array}{c} y=W⊛x+b\\ s=BN(y)\\ h=ReLU(s) \end{array}$$

(where ⊛ is the convolution operator).

They are followed by a global average pooling layer and a softmax. The batch normalization is used to improve generalization [32] and performance, together with the dropout layer, while the global average pooling (instead of a fully connected layer) reduces the number of parameters and, at the same time, the overfitting probability.

In this way, the FCN is used to extract the features and pass them to the global average pooling layer and the classification performed by the softmax. The net architecture was developed in Python and can be seen in Table 2, where the summary obtained using Keras is reported, which was used as an interface for the TensorFlow library.

The training dataset was prepared as described above (Section 3.1) and the 40,000 sequences were fed into the network. The number of peaks for the active/non-active state were randomly selected in two different ranges, whose values were set as in [6]. Moreover, the synthetic sequences were divided into training and validation ones. We used 80% of data as the training set and 20% as the validation set. As a metric to evaluate the model, the sparse categorical accuracy was chosen and the optimized was Adam.

## 5. Data Analysis and Results

After the training phase using synthetic data, the network was tested on both synthetic and experimental data as illustrated in the following.

**Figure 10:**
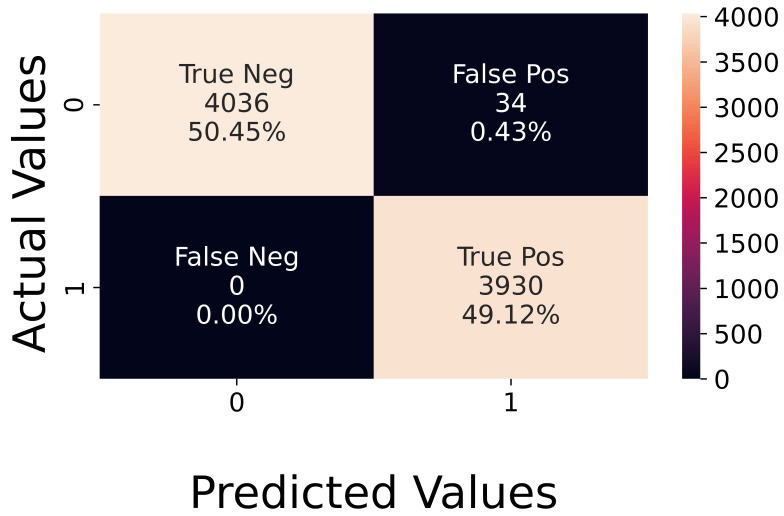
Confusion matrix with synthetic test data.

**Analysis of Synthetic Data.** The network was first tested using synthetic data, reaching an accuracy of 96%, with a loss of 0.14. Figure 10 shows that the network makes errors only on some neutral sequences, which are detected as active. The number of false positives is low, and the accuracy is balanced on both classes. This high accuracy value confirms that using a global average pooling layer, instead of a fully connected one, was enough to prevent overfitting, as explained in Section 4.

**Figure 11:**
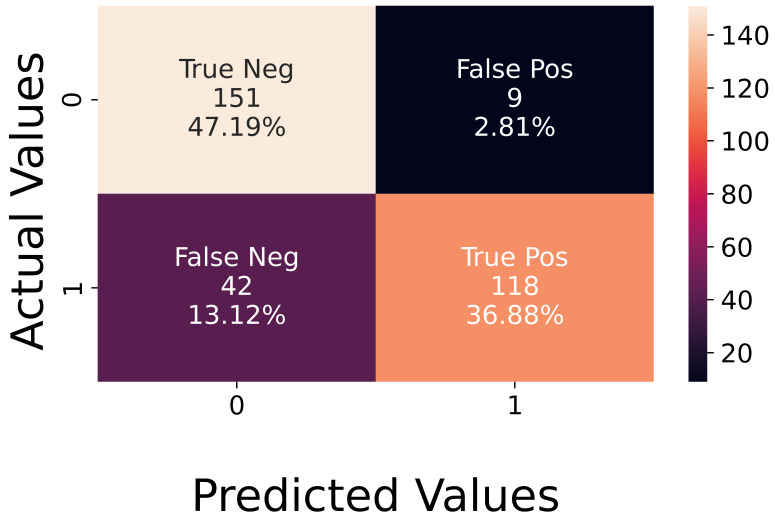
Confusion matrix on experimental data.

**Analysis of Experimental Data.** The recorded experimental dataset is balanced since it is composed of 160 active sequences and 160 neutral ones. The achieved accuracy was 84%, and the corresponding confusion matrix is shown in Figure 11. The figure illustrates that the network does not easily recognize the active sequences, which did not occur for synthetic data. In the latter case, the error was slightly higher on neutral sequences.

The expected different behavior of the network when testing synthetic or experimental data [12] can be due to our data generation model. Indeed, in future works, we aim at improving the model by taking into account more variables that have a relevant influence on the emotional state of the person.

**Figure 12:**
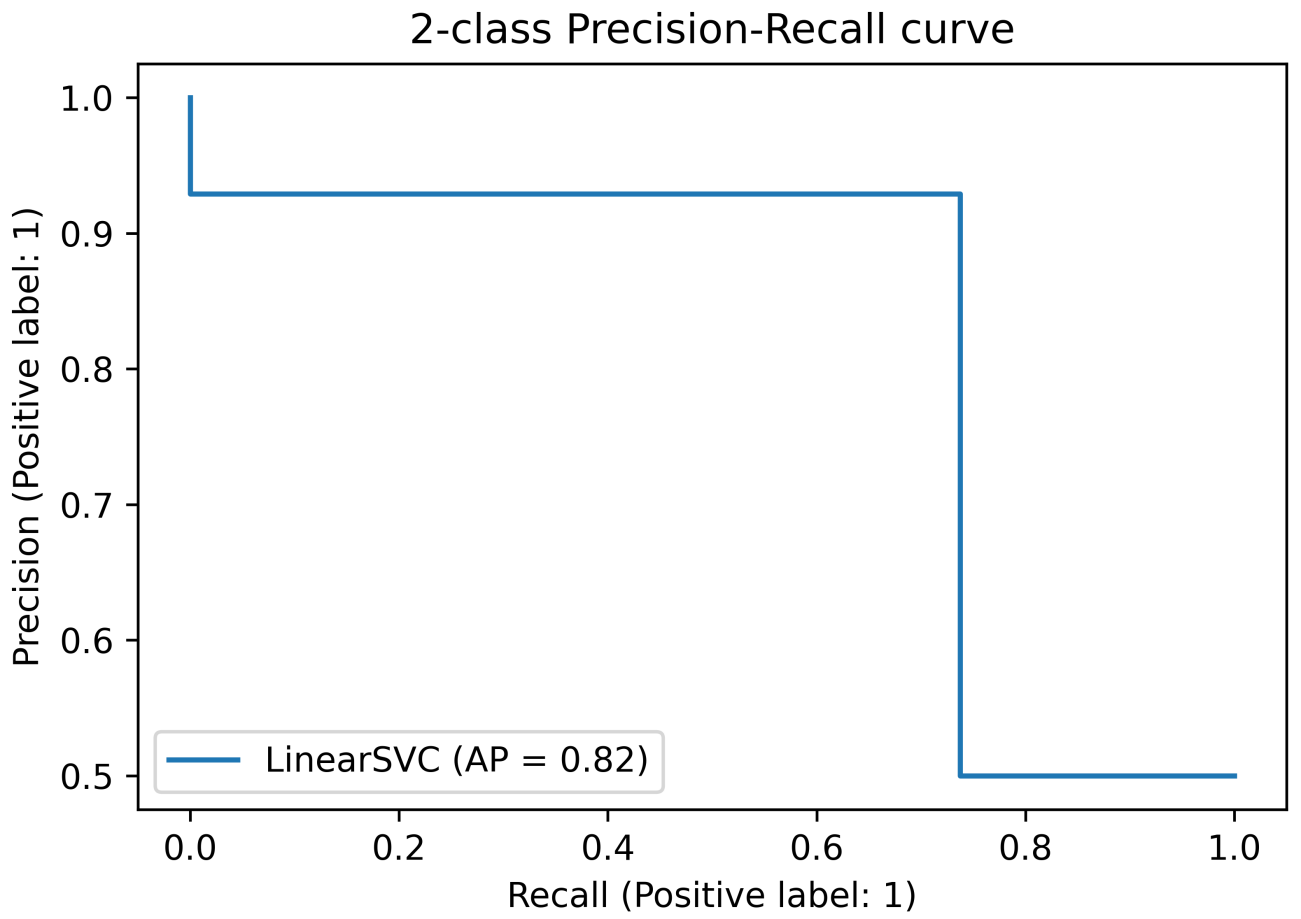
Precision–recall curve on experimental data.

Figure 12 shows the precision–recall curve obtained with the experimental data. The recall is 0.93, while the precision is 0.74. This means that the network does not miss any negative (non-active state) while it misses some of the positives (active states).

It is worth noting that the goodness of the results is application-dependent. Specifically, the method can be used as a complementary tool to deal with stress, or to let caregivers know if medication is needed to prevent a crisis. In the latter case, it would be better to avoid unnecessary medication, and this would be guaranteed by the high recall obtained with the network. On the contrary, if the developed tool is employed alone for the overall stress assessment, neglecting other warning signs, an unpredicted crisis could happen. Anyway, a good compromise is reached by the proposed method since, even if some anomalies are missed, it is highly desirable to avoid unnecessary potentially detrimental medication.

**Figure 13:**
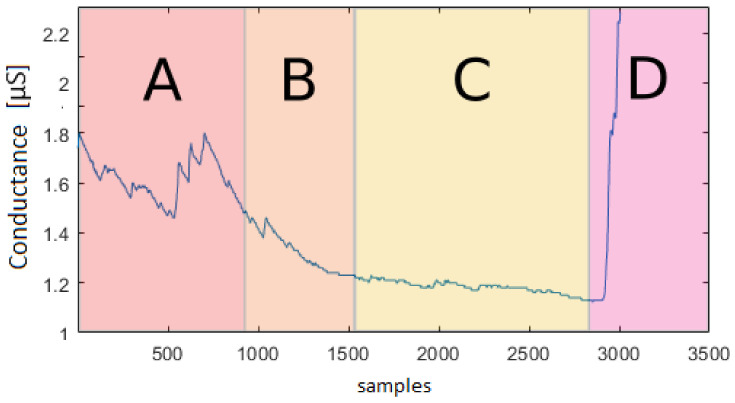
Conductance [μS] vs. number of samples (total sequence length—10 min; recorder with 5 Hz frequency): EDA signal during the relaxing state. In segment *A*, the meditation starts; in segment *B*, the signal intensity decreases; in *C*, there is an absence of peaks, and the signal is almost constant; in *D*, the meditation ends with a consequent signal intensity increasing.

Figure 13 shows a recording underlying the different levels of active/non-active state during a relaxing mode. This confirms that further quantitative studies of the EDA signal characteristics are needed to better model the data. In this way, a precise synthetic data model could replace the current need of extensive experimental datasets. Moreover, in Figure 13, it is possible to see how, at the very beginning, the subject is not relaxed yet, and the previous activity had an influence on the relaxation task. It is important to understand the influence of stimuli on people and its duration to simulate it more accurately.

## 6. Conclusions and Further Developments

A novel method to classify EDA signals was developed, to allow a fast understanding of the emotional state for non-verbal people, using a DNN. An EDA signal model was developed to generate synthetic data, which were used for the first time, as far as the authors know, for NN training purposes. We generated training sequences to represent the neutral and the active states, by better modelling both the number and the amplitude of SCR peaks. The achieved accuracy was 84% on the experimental data and 96% on the synthetic ones, demonstrating the feasibility of the proposed approach.

Future works will focus on the optimization of EDA model parameters setup and on modelling individual differences by distinguishing not only the activity level but also the valence of the arousal to gain information about the subject’s comfort. Moreover, we will improve noise analysis and filtering of experimental data by preprocessing techniques. Another important task would be the recording and analysis of sequences from several autistic subjects with different severity levels of autism, since a different neurology has to be taken into account if a psychophysiological signal is taken into consideration.

Other future research directions include the development of compressive sensing methods to reduce the complexity of the classification solutions [33], as well as the usage of multiple wireless battery-powered devices for higher performance and comfort, where it is essential to develop opportunistic wake-up techniques with location awareness of devices [34,35] to minimize the energy consumption as required in body area networks.

## Figures and Tables

**Table 1:** Comparison of different EDA classification techniques. These classifications are made considering arousal and not valence.

Paper	Accuracy	Year
CNN + SVM [21]	74%	2021
CNN + Decision Tree [21]	70%	2021
CNN + LDA [21]	72%	2021
CNN + MLP [21]	71%	2021
CNN [17]	85%	2019
ANOVA + SVM [3]	89%	2017
SVM-RFE [11]	94%	2021
SVM [19]	86%	2021
Fisher projection and LDA [20]	82%	2018
TSD + LSVM [22]	77%	2018

**Table 2:** New net architecture (Keras notation).

Layer (Type)	Output Shape	Param #
input 4 (InputLayer)	[(None, 600, 1)]	0
conv1d 9 (Conv1D)	(None, 600, 64)	256
batch normalization 9 (BatchNormalization)	(None, 600, 64)	256
re lu 9 (ReLU)	(None, 600, 64)	0
conv1d 10 (Conv1D)	(None, 600, 64)	12,352
batch normalization 10 (BatchNormalization)	(None, 600, 64)	256
re lu 10 (ReLU)	(None, 600, 64)	0
conv1d 11 (Conv1D)	(None, 600, 64)	12,352
batch normalization 11 (BatchNormalization)	(None, 600, 64)	256
conv1d 11 (Conv1D)	(None, 600, 64)	12,352
re lu 11 (ReLU)	(None, 600, 64)	0
global average pooling1d 3 (GlobalAveragePooling1D)	(None, 64)	0
dense 3 (Dense)	(None, 2)	130

## Data Availability

Not applicable.
